# A review of optical and digital aids for magnification techniques in low-vision rehabilitation

**DOI:** 10.14440/jbm.0002

**Published:** 2025-08-22

**Authors:** Mutali Musa, Ehimare Enaholo, Babatunde Ismail Bale, Fabiana D’Esposito, Caterina Gagliano, Rosa Giglio, Marco Zeppieri

**Affiliations:** 1 Department of Optometry, Faculty of Life Sciences, University of Benin, Benin, 300283 Edo, Nigeria; 2 Africa Eye Laser Centre, Benin, 300283 Edo, Nigeria; 3 Centre For Sight Africa, Nkpor, 434105 Anambra, Nigeria; 4 Imperial College Ophthalmic Research Group [ICORG] Unit, Imperial College, NW1 5QH London, UK; 5 Department of Medicine and Surgery, University of Enna “Kore”, 94100 Enna, Italy; 6 Eye Center G.B. Morgagni-DSV, 95125 Catania, Italy; 7 Department of Medicine, Surgery and Health Sciences, University of Trieste, 34127 Trieste, Italy; 8 Department of Ophthalmology, University Hospital of Udine, 33100 Udine, Italy

**Keywords:** Low vision, Magnification techniques, Optical aids, Electronic aids, Assistive technology

## Abstract

**Background:**

Low vision, a condition characterized by significant visual impairment, poses considerable challenges to individuals’ daily functioning and quality of life. Magnification techniques play a pivotal role in mitigating these challenges by enhancing visual acuity and enabling better access to printed materials, digital interfaces, and environmental cues.

**Objective:**

This paper provides a comprehensive overview of magnification strategies employed in low-vision rehabilitation. The review encompasses optical aids, such as magnifiers, telescopes, and microscopic devices, as well as electronic aids, including closed-circuit televisions, screen magnification software, and portable handheld devices. In addition, it explores the integration of magnification techniques with other assistive technologies and adaptive strategies to optimize functional vision. Furthermore, the article discusses emerging trends in magnification technology, including advancements in digital image processing, augmented reality, and wearable devices, which hold promise for further enhancing accessibility and independence for individuals with low vision.

**Conclusion:**

Understanding the diverse array of magnification options and their applications is crucial for eye care professionals, rehabilitation specialists, and individuals with low vision, enabling them to effectively navigate the visual challenges associated with this condition and promote greater inclusion and autonomy in daily activities.

## 1. Introduction

Low vision is defined as a condition in which the vision in the better eye is <20/60 after standard correction, or a visual field of <20% from the point of fixation.^1^,^2^ The National Health and Nutrition Examination Survey^3^ estimated that the prevalence of low vision in the United States was 1.1% in people aged 45 years and above, using a cutoff visual acuity of 6/18. Canadian optometrists also self-reported that approximately 1% of their patients were low-vision patients.^4^ Yekta *et al*.^5^ analyzed prevalence rates from 80 articles drawn from all over the world in individuals under 20 years of age and reported an average index of 1.67% (95% confidence interval [CI], 0.97–2.37%).

The majority of low-vision patients present in the latter stages of ocular morbidity, with males being more likely to visit the low-vision clinic.^6^ There is also a gap between the availability and uptake of low-vision services worldwide. The underrepresented elderly population is a focus of research attention due to their increased vulnerability to age-related visual impairment.^7^ Consequently, their daily activities are affected due to their diminished vision.^8^ The most prevalent functional challenge reported by individuals with low vision is difficulty with reading, and enhancing reading ability often serves as the primary objective of vision rehabilitation interventions, with the potential to positively impact cognitive function.^9^ Through low-vision aids and rehabilitation, patients regain the ability to carry out their daily living tasks by utilizing appropriate optical devices and making necessary environmental modifications to accommodate their residual vision.^10^,^11^ Hence, when selecting appropriate low-vision aids for visual rehabilitation, consideration is typically given to factors such as age, current visual acuity, disease progression, duration, level of education, and occupation.^12^

Furthermore, alongside the use of low-vision aids, supplementary reading training can enhance reading speed and overall quality of life.^13^ For example, Dickinson *et al*.^14^ showed that remote training of individuals suffering from age-related macular degeneration (ARMD) reported a significant improvement in the quality of life and near visual acuity after receiving training. On the other hand, patients with low vision are also more likely to complain about balance and musculoskeletal problems compared to a similar, normally-sighted demographic.^15^

## 2. Importance of magnification in low-vision rehabilitation

Magnification helps to increase both the retinal image size and the angle subtended at the higher visual center. The use of magnification proves to be a beneficial approach in the rehabilitation of individuals with low vision, demonstrating favorable clinical outcomes while also being economically efficient.^16^,^17^ However, Thomas *et al*.^18^ reported a paucity of data on the effectiveness of magnification and low-vision therapy in all age demographics. Nevertheless, when providing glasses and magnifiers for visual rehabilitation to enhance quality of life, it is crucial to explore the financial implications of the intervention for all major stakeholders, including patients, families, and professionals.^19^ This consideration is important for patients, as it could potentially improve their visual capabilities.

A study conducted by Latham and Macnaughton^20^ reported that identifying the print size that low-vision patients find comfortable for reading can serve as an effective indicator for estimating the magnification needed in their rehabilitation process. It is generally more effective to assess and determine their reading speed and critical print size using single sentences rather than paragraphs, except in situations requiring repeated measurements, such as tracking the progression of a reading disorder or evaluating intervention outcomes.^21^ Granquist *et al*.^22^ showed that low-vision patients hold prints at closer distances and use larger prints subjectively when reading words as compared to normal-sighted individuals. However, for consistent results, paragraphs are preferred due to their reduced variability. It is important to note that improving reading skills significantly enhances occupational performance, daily activities, and social involvement among the elderly population with low vision.^23^ To achieve this, occupational therapy professionals should incorporate the following interventions into their standard care: (i) utilizing stand-based electronic magnification; (ii) providing eccentric viewing training; and (iii) offering comprehensive low-vision services.^24^

This paper reviews the studies on magnification in low vision published within the past decade to examine the history and innovations in the field. The principles and characteristics of magnification systems are discussed, and the advent of technology in low-vision care is also reviewed.

## 3. Methodology

**Figure 1:**
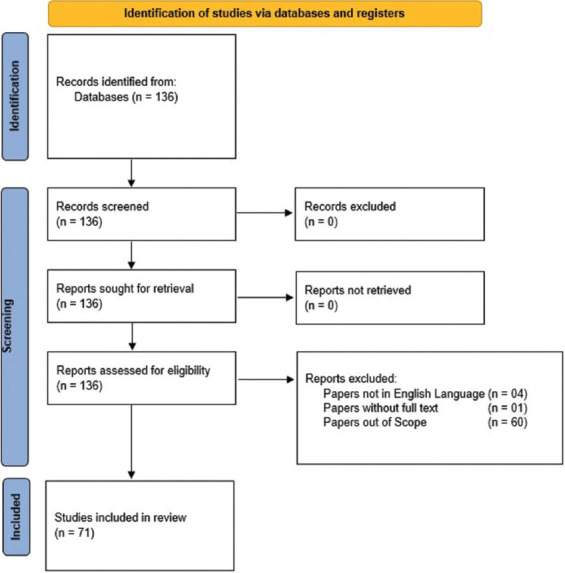
Preferred Reporting Items for Systematic Reviews and Meta-Analyses flow diagram for the selection process of papers

The authors used the term “Low-vision magnification” as a search query on the PubMed, reference citation analysis, and Scopus databases. The search algorithm for PubMed was “((“vision, low”[MeSH Terms] OR (“vision”[All Fields] AND “low”[All Fields]) OR “low vision”[All Fields] OR (“low”[All Fields] AND “vision”[All Fields])) AND (“magnification”[All Fields] OR “magnifications”[All Fields])) AND (2014:2024[pdat]).” All retrieved articles were subsequently screened for relevance and formatted by two of the authors (M. Musa and B. Bale). Articles were excluded if they met any of the following criteria: not written in English, lacking a full text or abstract, or irrelevant to the topic. Additional exclusions included articles that did not provide peer-reviewed full text, such as unpublished abstracts or non-peer-reviewed conference proceedings, as well as duplicate entries. Both open-access and subscription-based articles were evaluated to determine if they provided complete content accessible to authors through institutional access or interlibrary loan. A total of 65 papers were thereby excluded, while the remaining 71 papers were reviewed in this paper. A preferred reporting items for systematic reviews and meta-analyses^25^ chart showing these search criteria is shown in Figure 1.

## 4. Optical magnification techniques

### 4.1. Primary magnification techniques

There are three major magnification techniques utilized in low-vision care^26^;

#### 4.1.1. Relative size magnification

**Figure 2:**
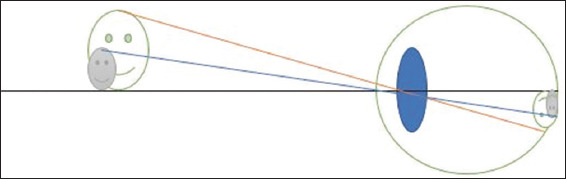
A schematic representation of relative size magnification showing how a bigger object results in a bigger image size. The eyeball is depicted as the large circle surrounding the lens, and the retina is represented by the outline of the eye in the posterior section. The object is the smiley face on the left side of the image. This represents the external object being viewed. The incident light rays are indicated by the orange and blue lines, which project from the object toward and then enter the eye. The blue oval structure in the middle of the eye and the green line in front represent the optical components (cornea and lens) that refract light. The retina (image plane) is shown as the inner surface at the back of the eyeball, where the light rays converge and form an inverted image. The image on the retina is represented as a small, inverted smiley face, which represents the focused image of the object.

Here, magnification is achieved by increasing the size of the object viewed, thereby achieving a bigger retina image (Figure 2). This is akin to using a large-print book as opposed to its normal-sized print counterpart. The magnification produced can be derived from Equation I.


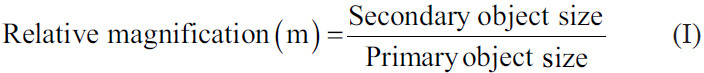


#### 4.1.2. Relative distance magnification

**Figure 3:**
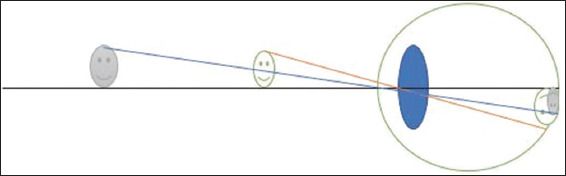
A schematic representation of relative distance magnification showing how a closer object results in a bigger image size. The eyeball is depicted as the large circle surrounding the lens, and the retina is represented by the outline of the eye in the posterior section. The object is the smiley face on the left side of the image. This represents the external object being viewed. The incident light rays are indicated by the orange and blue lines, which project from the object toward and then enter the eye. The blue oval structure in the middle of the eye and the green line in front represent the optical components (cornea and lens) that refract light. The retina (image plane) is shown as the inner surface at the back of the eyeball, where the light rays converge and form an inverted image. The image on the retina is represented as a small, inverted smiley face, which represents the focused image of the object. The grey color indicates that the object is not sharply focused on the retina. The eye is instead focused on the middle green smiley face, which has rays converging perfectly onto the retina.

Magnification is achieved by bringing the object of regard closer to the patient, making it larger and easier to see (Figure 3). The drawback of this method is that large amounts of accommodation must be present or compensated for with high plus lenses to account for the short working distances. This can be derived from Equation II.


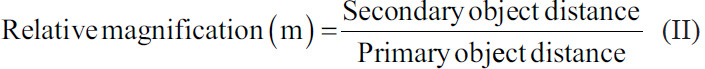


#### 4.1.3. Angular magnification

**Figure 4:**
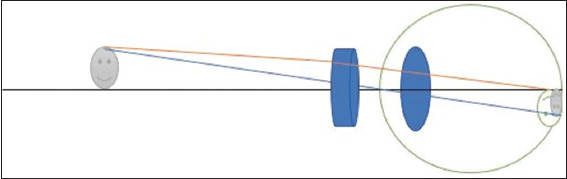
A schematic representation of angular magnification showing how a lens creates a larger angle of resolution and a bigger image size. The use of a corrective convex (plus) lens placed in front of the eye can compensate for a hyperopic (farsighted) optical system. In the uncorrected state, parallel rays from a distant object (represented by the blue line from the grey smiley face) would converge to a point behind the retina, resulting in a blurred retinal image. The corrective lens redirects the incoming light rays so that they are refracted more strongly before entering the eye, allowing them to focus directly on the retinal surface. The grey smiley face represents a distant object that would normally appear out of focus in a hyperopic eye. The additional blue lens in front of the cornea represents a plus lens used for hyperopia correction (*e*.*g*., glasses or contact lenses). The orange line depicts how light is refracted by the corrective lens and then further focused by the cornea and crystalline lens. The sharp, inverted image on the retina indicates the correction of refractive error.

Here, the object of view is not brought closer, nor is its size magnified, but the angle subtended by the image produced by the object is magnified, allowing the higher visual centers to see the target (Figure 4). This can be derived from Equation III.


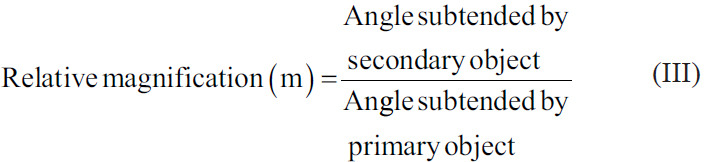


### 4.2. Magnification determination and examples of magnifiers

The magnification required to achieve a task is shown in Equation IV.


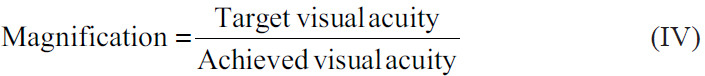


Hence, a patient who sees a final corrected visual acuity of 6/36 and intends to see the 6/9 line will need a 4 × telescope. The same applies to near work, where a patient who wants to read 1 M but has a final corrected visual acuity at near 6 M will need a magnification of 6 ×. Some magnifiers are calibrated in diopters, as opposed to magnification power. However, these can be cross-converted using Equation V.^27^,^28^


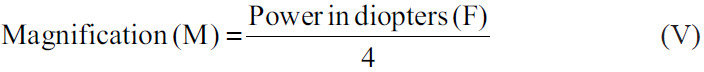


In determining near magnification, Kestenbaum’s rule is also sometimes applied. This rule states that the reading add is simply the reciprocal of the distance visual acuity.^29^ Another study by Engesser *et al*.^30^ has suggested that when compared to a final low-vision prescription, clinical records alone cannot correctly estimate the amount of magnification required by a patient. Some magnifiers are displayed in Table 1.

**Table 1:** Magnification devices used in low vision

Type of magnifier	Advantages	Disadvantages	Distance used	Image
Spectacle magnifier^31^	-Hands-free - Socially acceptable	-Proximity of prints and tasks to the face during use. - Excessive weight of spectacles	Near	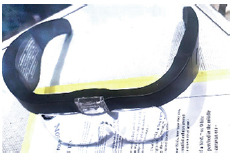
Handheld magnifier^32^	-Socially acceptable -Adjustable magnification - Suitable for domestic tasks	-Limited applicability for individuals with hand tremors	Near	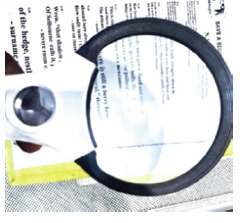
Stand magnifier^33^	-Fixed object distance - Constant magnified image	-Fixed magnification - Not suitable for writing tasks	Near	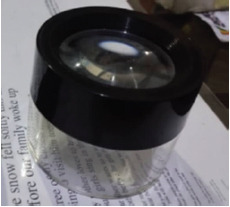
Telescope^34^	-Variable focus - Applicable as a field expander (reverse telescope)	-Relatively greater expense than other aids	Distant and near (with a reading cap)	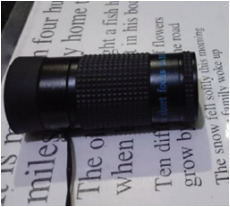
Hi-tech aids^35^	-Applicable as a mounted device -Head-mounted device with improved reading speed^36^ -Adjustable contrast to aid vision^37^	-Excessive cost	Distance and near	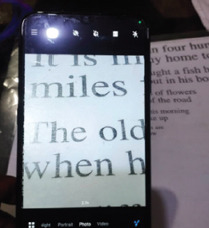

#### 4.2.1. Magnifiers

Magnifiers are typically high-power lenses or systems that help increase the perceived size.^37^ They operate on the principle of relative size magnification. They can be spectacle magnifiers, handheld magnifiers, stand magnifiers, and telescope magnifiers. The magnifiers can be illuminated or non-illuminated. It should be noted that stand magnifiers are used in conjunction with reading lenses to enable the user to focus the divergent rays that are emitted from this aid.

Multiple factors affect the selection of magnifiers for patients. Particularly, the magnification required by the individual patient, as well as the distance at which this magnifier is to be used. While Table 1 lists several magnifiers for both far and near distances, there is a demographic that requires magnification aids effective at both distances simultaneously. Ambrogi *et al*.^38^ fabricated a device that facilitated this by capturing distant images and projecting them onto a screen close to the patient, while also magnifying near work. Afinogenov *et al*.^39^ also designed a device prototype that enabled magnification of both distant and near targets up to 8 times the normal.

The variance of tasks required by low-vision patients leads to the dispensing of a combination of aids/devices after the low-vision assessment. Gobielle *et al*.^40^ reported that low-vision patients may, however, abandon low-vision aids after dispensing, concluding that 29% of them stop using at least one optical assistance within 3 months. One possible reason for this may be the constricted field of view produced by many low-vision devices.^41^

Employing magnification as an aid for individuals with low vision enhances their best-corrected visual acuity, both at a distance and near, and also improves their stereopsis. However, the improvement at a distance is not statistically significant.^42^ Johnson *et al*.^43^ pointed out that social interaction by low-vision patients is hampered by their inability to perceive emotions on the faces of others. Their study, however, concluded that magnification alone will not improve the ability to categorize facial expressions of emotion. Gaze-contingent low-vision aids have been shown to statistically enhance facial recognition and response time in patients with low vision and central field loss, from 41% to 63% (95% CI).^44^

Even with the advent of modern assistive technologies and electronic magnifiers, classic optical magnification remains a boon to low-vision rehabilitation, including activities of daily living (ADLs) performed at near distances.^45^ Telescopic magnifiers, such as the bioptic, have also been useful in driving.^46^ Care should be taken, however, when driving with the bioptic, as it may also affect the visual field due to the ring scotoma created by the telescope.^47^ The cost of the aid also influences the type of magnifier that patients require. Kyeremeh and Mashige reported that the high cost of low-vision aids was the second-highest barrier preventing the utilization of these aids.^48^

#### 4.2.2. Telescopic systems

**Figure 5:**
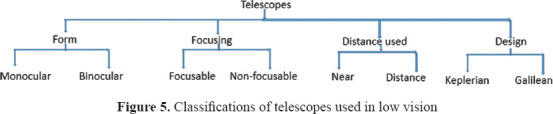
Classifications of telescopes used in low vision

Telescopes operate on the principle of angular magnification to bring distant objects into the user’s view. Telescopes can be generally classified as either Keplerian or Galilean.^49^ Keplerian telescopes have plus lenses as the objective and ocular lenses. Galilean telescopes, on the other hand, have a minus lens as the objective with a plus lens as the ocular. Telescopes can be classified according to multiple factors, as shown in Figure 5.

Telescopic vision aids are mostly indicated for rehabilitating ADLs performed at a far distance.^46^ However, their use can be modified for near function by including the near addition lens power as a focusing cap.^27^ Monocular telescopes inhibit proper flat fusion; hence, they are not suitable for dynamic tasks. Novel intraocular, implantable, and miniature telescope designs have been associated with good functional adaptation following rehabilitation among patients with marked unilateral central vision impairment/loss.^50^ Bioptic telescopes adapted for specific targets, such as street/road signs, can be more suited to dynamic activity. Galilean telescopes are used for low-vision rehabilitation due to their lower magnification capabilities and subsequent suitability for visual adaptation.^51^ High magnification telescopes restrict the total field of view range, and their longer tube lengths can compromise design acceptability.

In albinistic low-vision patients, nystagmus can be a significant problem, causing visual deficits when the eye moves across the surface of a thick lens, as is often the case with handheld, stand-mounted, and spectacle magnifiers.^52^ Although authors have recommended surgery as a palliative measure,^53^ telescopes are a viable option to improve vision at near distances. Dysli and Abegg^54^ have dissented from this, suggesting that other sensory impairments, such as visual acuity, may be responsible for reading difficulties in people with albinism, rather than nystagmus. Their study found comparable reading speeds when the words were large enough and moved parallel to nystagmic saccades, as observed when comparing people with albinism to healthy subjects.

#### 4.2.3. Microscopic devices

Telemicroscopes are special spectacles equipped with a primary lens that incorporates extra lenses, combined with the near portion.^55^ They can also come as bioptics, where an additional lens is mounted in the distance portion over a primary lens. Telemicroscopes are particularly useful when a patient requires magnification at one distance while maintaining appreciable vision at other distances.^56^ It is therefore used for tasks such as crafting, inspection, and spot distance viewing. Telemicroscopes are more socially acceptable than telescopes. They are also lighter and easier to use as there is no need for physical handling. This also means that elderly patients with tremors can benefit from them. They are ideal for patients who switch between distance and near vision during their daily activities.

The use of contact lenses in low-vision magnification has gained traction in recent years. Contact lenses offer better weight considerations and cosmetics, in addition to being useful for glare control in patients with albinism and low vision.^57^ Vincent^58^ suggested that the contact lens forms the eyepiece of a telescopic system, while a spectacle lens worn over the eye serves as the objective. Matchinski *et al*.^59^ reported on a case where they used a contact lens as a reading cap, paired with a telescope, to enable a patient to read near print. Theoretically, this can also increase the field of view available to the patient and may be particularly beneficial in patients with already constricted visual fields.^57^

#### 4.2.4. Electronic magnification aids

Electronic magnification aids help magnify objects without the need for physical lenses.^60^ This frees the user from any manipulation or physical activity when using them. The most common type of electronic magnification aid used in low-vision care is the closed-circuit television (CCTV).^36^ The information generated by the CCTV is not transmitted but rather remains confined to the device itself, ensuring user privacy. The equipment typically consists of an inbuilt camera, an image processor, and a screen.^61^ CCTVs offer extended magnifications of up to 100×, reverse polarity, and illumination controls.^62^ One major drawback of CCTV is that its use is difficult to learn, which is further complicated in patients with low vision.^63^

Individuals with corneal diseases necessitating visual rehabilitation through low-vision aids experience improved reading speed and performance when using CCTV against a dark background, likely due to its ability to minimize luminescence, as suggested by research findings.^64^ Magnification alone may not be sufficient to help every low-vision patient, and activities such as preferential-looking techniques, eccentric fixation, and oculomotor training can be helpful.^65^ Clinicians should ensure that patients participate actively when training to achieve the best results.^66^

### 4.3. Role of smartphones and tablets in magnification

With the emergence of smartphones, many daily tasks are now completed on the go using these handheld devices. Da Silva *et al*.^67^ assessed the usability of free magnification apps on the reading characteristics of low-vision patients and concluded that every free app sampled improved reading speed and visual acuity. Conversely, the magnification feature in smartphones has proven beneficial for basic microsurgery training. Nonetheless, its application for this purpose faces challenges regarding its three-dimensional visualization and overall visual clarity.^68^ Numerous software applications are available that can be useful for screen magnification on handheld devices. Some of these are native to the device, while others must be installed. One problem of using the software applications is that the user sometimes gets lost and cannot retrace their view to the initial starting point due to the constricted field of view that comes with magnification. Zoomtext is a software application that enables users to magnify images and print on their devices, while also allowing them to lock scrolling horizontally or vertically, making it easier to return to the starting point.^69^ An additional cutting-edge assistive technology for low-vision rehabilitation is the ArtontheBrain application, which holds promise in making visual art accessible and attainable for leisure, recreation, and therapeutic interventions tailored to individuals with low vision.^70^ Luo^71^ developed a smartphone-based magnification app embedded with a sensor algorithm to monitor its use. Activity from over 16,000 individuals from more than 120 states was logged in the study, and the data revealed that the app was used for <3 min a day. A possible reason for this may be the lack of training and awareness of these magnification capabilities among patients who use them.^72^

Digital devices possess multiple advantages over optical aids, including:

(i) Digital devices can manipulate the characteristics of the image projected without physically altering the object. Users can invert colors, adjust shade and hue, or selectively highlight text, all to maximize their vision. 62
(ii) Digital devices can offer variable magnification even above and below values that may be out of the limits for standard magnifiers. 73
(iii) Digital devices are more socially acceptable than standard optical aids. 74

Luo’s^71^ study suggested that common problems associated with the use of low-vision apps on smartphones included image shaking caused by users’ weak grip, which he recommended solving with image stabilization. Morrice *et al*.^75^ further suggested that mobile handheld devices are reasonably comparable to CCTV and other digital devices. In general, smart devices, such as the iPad, have been investigated for their ability to perform comparably to specially designed video magnifiers.^76^ There are, however, valid concerns that zooming the text on smartphones can greatly restrict the field of view, causing a loss of context for the information being read.^77^ Pundlik *et al*.^78^ piloted a solution to this when they used Google Glass to remotely access the screen of a smartphone, thereby increasing the magnification of texts, and rather than having to zoom in on the handheld and see a few words, users could pan their head from side to side to read the whole text string at once as akin to a book.

## 5. Integration with assistive technologies

Leveraging smartphones and applications as assistive tools offers magnification and zoom capabilities to aid individuals with low vision, while also facilitating text input and output, as well as command execution through speech features such as Siri and Talkback, which are particularly beneficial for those who are blind.^79^ Because individuals with low vision often encounter challenges when using screen magnifiers to navigate and interact with productivity tools, MagPro presents itself as a user-friendly application interface enhancement, offering an alternative technology that markedly reduces the effort required for panning and zooming.^80^

## 6. Strategies for maximizing functional vision through technology integration

In a recent preliminary investigation conducted by Bittner *et al.*,^81^ it was found that the reading skills and efficiency of individuals with low vision can be enhanced by utilizing tele-rehabilitative technology for training with new magnifiers (including handheld magnifiers, stand optical magnifiers, and portable electronic magnifiers) as opposed to traditional in-office training. Visually impaired users found this videoconferencing technology feasible and acceptable.^82^ Telerehabilitation offers the option for remotely assessing low-vision services instead of in-office training with new magnifiers.^83^ Novel laser eyewear demonstrated good potential for optimized augmented vision through direct retinal image projection for individuals with disorders affecting the corneal media transparency.^84^

## 7. Advancements in digital image processing

In clinical applications, remote sensing aided by magnification is currently reported to have advanced to the point where physiological indices, such as heart rate and respiratory rate, can be measured at a distance from a patient.^85^ This potentially means that low-vision patients may accurately assess these important measures in family and loved ones, even in the presence of visual deficits. While the Bubble magnification technique, an electronic magnification method that enlarges a focal area based on gaze direction, can mitigate resolution and crowding issues, it does not enhance the video comprehension of individuals with central vision loss.^86^ Billah *et al*.^87^ also trialed the SteeringWheel software for helping low-vision patients browse through web pages without losing context due to the magnification of a few words at a time. The software allowed the sampled subjects to simplify the complex browser screens into sections that can be rotated and previewed before being clicked for magnification. Functional vision in reading and visual information processing is enhanced by innovative approaches in magnification, specifically the virtual bioptic telescope and virtual projection screen, which utilize digital image processing within a head-mounted display.^88^

## 8. Future directions for enhancing accessibility and independence in individuals with low vision

Rehabilitation success depends not only on the choice of suitable magnification equipment but also on organized training programs that facilitate optimal utilization of this equipment. Rehabilitation programs should ideally include task-specific practice, education in eccentric seeing skills, and tactics for contrast enhancement. Moreover, interdisciplinary cooperation among optometrists, occupational therapists, and vision rehabilitation specialists is crucial for customizing interventions that meet the individual’s vocational and social needs. Training must prioritize goal-setting, incremental device integration, and practical simulations to improve skill transferability.

Zhao^89^ suggests that augmented reality (AR) goes beyond traditional magnification, which can be compromised by a constricted field of view and aberrations, and provides low-vision patients with cues that help them achieve daily tasks, such as climbing staircases or navigating crowded spaces. It is crucial to note that AR and wearable magnification devices contain an element of minification for distance vision and magnification for near vision. McLean *et al*.^90^ showed that all minification levels, even as small as 2%, presented with significant discomfort, with no predilection for laterality. Potential users of wearable electronic magnification systems capable of varying angular size magnification may be discouraged from proceeding with device trials due to the cumbersome appearance of such head-mounted units.^91^ It may be worth customizing such vision aids for functional use when performing home-based tasks rather than during public appearances.^91^ Table 2 summarizes some articles on the functionality of wearable magnification devices in individuals with visual impairment.

**Table 2:** Summary of wearable devices for patients with low vision

Author	Device	Type of study	Sample	Outcome
Miller *et al.*^92^	Wearable electronic vision enhancement systems (wEVES)	Randomized controlled trial	32 individuals	wEVES gave image enhancement and better visual acuity
Gopalakrishnan *et al.*^93^	Augmented and virtual reality devices	Original research	100 individuals	Visual acuity and visual field expansion were improved in the sampled population.
Visser *et al.*^94^	E-scoop spectacle lens	Randomized controlled trial	190 individuals	The E-scoop spectacle lenses did not yield significant clinical benefits in terms of improving the quality of life, visual acuity, and contrast sensitivity for patients with ARMD.
Cottingham *et al.*^95^	Smartphone-assisted head-mounted wearable aid	Original research	18 individuals	Variable magnification yielded favorable subjective improvements in the quality of life of children younger than the age of 10

Abbreviation: ARMD: Age-related macular degeneration.

## 9. Importance of an individualized approach in magnification selection

Several factors influence the acceptability and usability of magnification devices for individuals with low vision. Some of these factors include the patient’s age, the cause of low vision, visual acuity, and the patient’s motivation.^96^ Telescopes are more widely accepted in relatively developed societies compared to less developed communities, where the use of telescopes in public may be associated with stigmatization. The amount of magnification prescribed is also closely related to the condition causing the low vision. Patients suffering from diseases that affect the central visual fields tend to perform better with high magnifications. At the same time, those with peripheral vision conditions, such as glaucoma and retinitis pigmentosa, would likely tolerate lower magnifications. Regarding music enthusiasts, the eSight Eyewear, a head-mounted low-vision rehabilitation device, effectively addresses the magnification challenges encountered by individuals with low vision when reading musical notes.^97^ Its adjustable magnification and hands-free design make it uniquely suited for this task. In addition, factors impacting its usage in low-vision rehabilitation include standardized assessments of device-related quality of life, the absence of headaches associated with its use, and satisfaction with post-usage support services.^98^ The use of wearable electronic vision enhancement systems is known to be complicated by the narrow field rendered by the magnification produced. Researchers have now developed an algorithm to enlarge a part of the text being read while also maintaining a clear view of the surrounding print in fields as small as 10°.^44^

Pundlik *et al*.^99^ developed an optical character recognition-based software that enables users to search for keywords in text or images displayed on a smartphone screen. This allows users to zoom in on areas of interest without having to navigate through a cluttered scene. With ARMD being the most common cause of low vision, researchers have achieved better visual acuity using a specialized intraocular lens (IOL) designed to provide enhanced vision in the central 10° as compared to standard IOLs.^100^

## 10. Comparative effectiveness of magnification aids

Although several magnification aids show advantages for those with low vision, direct assessments of their functional effectiveness are scarce. A Cochrane Review by Virgili *et al*.^36^ demonstrated that electronic video magnifiers provide superior reading speeds compared to optical magnifiers; however, user satisfaction fluctuated according to cost and use. Likewise, randomized controlled studies conducted by Visser *et al*.^94^ assessed E-scoop lenses and demonstrated little improvement in quality-of-life metrics. In contrast, head-mounted digital magnification systems have demonstrated subjective enhancements in pediatric quality of life and improved visual functions in adults with ARMD.^93^,^95^ This variety in outcomes underscores the importance of matching device type with individual patient objectives and abilities.^101^

Although magnification tools offer advantages, any detrimental effects must be taken into account. High-powered telescopes and microscopes can induce ring scotomas, create visual distortions, or restrict peripheral awareness, concerns that are particularly significant during walking or driving. Digital devices, although providing freedom, may induce visual strain from screen glare and necessitate cognitive adjustment. Moreover, bulky or prominent wearable gadgets may impede sustained usage due to discomfort or societal stigma. Personalized risk-benefit assessment and user education are essential for alleviating these issues.

## 11. Conclusion

This paper provides a comprehensive review of the magnification techniques in the context of low-vision rehabilitation. We have explored both optical and electronic aids, discussing their respective advantages, limitations, and applications in various daily tasks. From traditional magnifiers to advanced electronic devices, the array of magnification options offers individuals with low-vision newfound opportunities for independence and engagement in everyday activities.

Moreover, the authors emphasized the importance of integrating magnification aids with other assistive technologies to maximize functional vision and enhance overall quality of life. By leveraging advancements in digital image processing, AR, and wearable devices, the future of low-vision rehabilitation holds promising prospects for further improving accessibility and autonomy for individuals with visual impairments.

Clinicians are advised to select magnification devices based on assessments of acuity, task-specific requirements, cognitive abilities, and user preferences. Systematic training, risk reduction, and continuous assistance are crucial for effective integration. The impact of emerging technologies, such as wearable digital magnifiers and AR systems, on improving functional outcomes must be evaluated through stringent clinical trials to establish evidence-based guidelines. Through continued research, innovation, and collaboration within the field, we can strive toward a more inclusive society where individuals with low vision can fully participate and thrive in all aspects of life.

## Data Availability

Not applicable.
